# Гормон роста — 30 лет клинической практики: прошлое, настоящее, будущее

**DOI:** 10.14341/probl13432

**Published:** 2024-02-27

**Authors:** И. И. Дедов, О. Б. Безлепкина, М. С. Панкратова, Е. В. Нагаева, Е. Н. Райкина, В. А. Петеркова

**Affiliations:** Национальный медицинский исследовательский центр эндокринологии; Национальный медицинский исследовательский центр эндокринологии; Национальный медицинский исследовательский центр эндокринологии; Национальный медицинский исследовательский центр эндокринологии; Национальный медицинский исследовательский центр эндокринологии; Национальный медицинский исследовательский центр эндокринологии

**Keywords:** гормон роста, низкорослость, СТГ-дефицит, синдром Шерешевского-Тернера, синдром Ларона, идиопатическая низкорослость, задержка внутриутробного развития

## Abstract

Эра рекомбинантных технологий, начавшаяся во второй половине XX века, позволила производить рекомбинантный гормон роста (рГР), необходимый для лечения низкорослости различного генеза. Настало время практически неограниченных возможностей получения рГР, что послужило стимулом для изучения эффективности и безопасности применения рГР, поиска оптимальных способов его использования и режимов дозирования. Многолетний опыт применения соматропина в клинической практике позволил получить данные об его эффективности прежде всего при соматотропной недостаточности у детей, изучить его действие на функциональное состояние различных органов и систем, расширить показания к использованию рГР.

## Abstract (en)

The recombinant technologies era, which began in the second half of the XX century, made it possible to produce recombinant growth hormone (rGH) necessary for the treatment of stunting of various genesis. The time of practically unlimited possibilities of rGH production has come, which served as a stimulus for studying the efficacy and safety of rGH application, searching for optimal ways of its use and dosing regimes. Many years of experience in the use of somatropin in clinical practice allowed us to obtain data on its effectiveness primarily in somatotropic insufficiency in children, to study its effect on the functional state of various organs and systems, and to expand the indications for the use of RGR.

Практически неограниченные возможности получения рГР послужили стимулом для изучения эффективности и безопасности терапии с его применением, поиску оптимальных способов его использования и режимов дозирования. Впервые рекомбинантный человеческий гормон роста (рГР) был синтезирован в 1981 г. биотехнологической корпорацией Genentech (Сан-Франциско, Калифорния) [1].

Ранее для лечения гипофизарного нанизма применялся экстрагируемый из кадаверных гипофизов человека гормон роста [2]. В 1975 г. Н.А. Зарубиной была опубликована первая отечественная монография на тему «Гипофизарный нанизм (клиника, диагностика, дифференциальная диагностика, лечение)», где был обобщен опыт лечения гипофизарных карликов. Однако использование гормона роста человека, полученного из трупных гипофизов, сопровождалось рядом ограничений и рисков, а развитие у пациентов болезни Крейтцфельдта-Якоба подвигло к поиску альтернативных способов получения ГР. Поэтому широкое применение в клинической практике ГР получил только в конце 80-х — начале 90-х гг. прошлого столетия, после того как рекомбинантные технологии позволили производить его в неограниченных количествах.

Гормон роста стал вторым рекомбинантным препаратом, который был введен в клиническую практику вслед за инсулином. Поскольку исчезли ограничения в производстве гормона роста, начался этап активных клинических исследований с целью оптимизации лечения в отношении дозировки, времени введения и длительности применения препарата. В последующие годы рГР был одобрен для лечения различных форм низкорослости, было доказано, что он улучшает как конечный рост, так и влияет на многие обменные процессы в организме, в том числе улучшает психологические и социальные параметры.

В настоящее время создано множество препаратов рГР по всему миру: Сайзен («Мерк-Сероно», Италия), Хуматроп («Эли Лили», США), Омнитроп («Сандоз», Австрия), Генотропин («Пфайзер», США), Нордитропин («Ново Нордиск», Дания), Джинтропин («Дженсайенс», Китай) и другие. В Российской Федерации первый опыт применения соматропина датируется началом 90-х гг. XX века [3].

В 2006 году в Российской Федерации был зарегистрирован первый отечественный препарат рекомбинантного гормона роста — Растан. Высокая эффективность и безопасность длительного ежедневного применения рГР доказаны многолетним опытом его применения.

Таким образом, благодаря своевременной диагностике и доступности этиотропной терапии дети, ранее обреченные быть карликами, достигают целевых показателей роста и вырастают до 160–180 см.

## ЭПИДЕМИОЛОГИЯ

По данным зарубежных источников, распространенность соматотропной недостаточности составляет 1–4 случая на 10 000 детей [4]. В Российской Федерации дефицит гормона роста составляет 1–2 случая на 10 000 детского населения. Распространенность соматотропной недостаточности в России за 9-тилетний период (2014–2022 гг.) составляет 17,5 на 100 000 детей и не одинакова в разных федеральных округах страны (рис. 1). Максимальная распространенность заболевания выявлена в Северо-Западном федеральном округе — 20,4 на 100 000 детей, меньше всего — в Сибирском (9,8 на 100 000 детей) и Южном федеральных округах (11,8 на 100 000 детей). Показатели встречаемости соматотропной недостаточности в Приволжском, Центральном и Дальневосточном федеральных округах близки к среднероссийским (15,2; 16,5 и 17,5 на 100 000 детского населения соответственно). В Северо-Кавказском и Уральском ФО заболевание встречается соответственно у 13,5 и 14,5 на 100 000 детей. Несмотря на территориальные различия имеется общая тенденция к увеличению распространенности заболевания во всех регионах Российской Федерации, в целом по стране распространенность за 9 лет увеличилась практически в 2 раза: с 8,8 до 17,7 на 100 000 детей.

**Figure:**
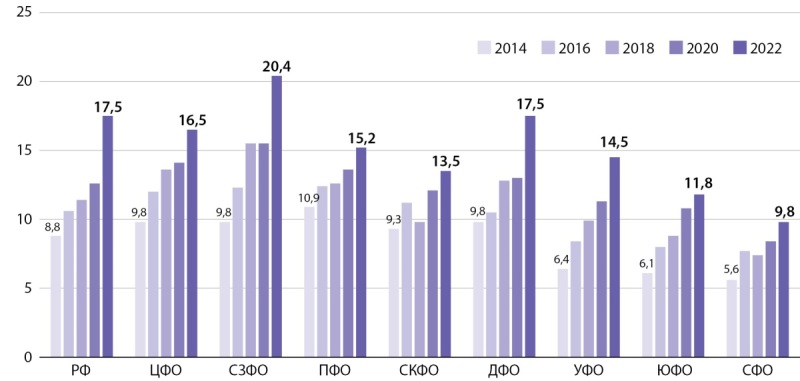
Рисунок 1. Динамика распространенности СТГ-дефицита в Российской Федерации на 100 000 населения по федеральным округам (2014–2022 гг.).

## СТГ-ДЕФИЦИТ У ДЕТЕЙ

Лечение СТГ-дефицита у детей является первым и основным показанием к заместительной терапии гормоном роста [5][6]. Длительные наблюдения подтверждают, что при своевременно установленном диагнозе у детей возможно достижение целевых показателей роста. Многолетний опыт лечения детей с соматотропной недостаточностью в Институте детской эндокринологии доказал эффективность и безопасность длительной терапии рГР на большой когорте российских пациентов. Детальный анализ длительной терапии гормоном роста 422 пациентов (202 девочки и 220 мальчиков) показал, что суммарная прибавка в росте составила у лиц мужского пола — 58,4 см, у лиц женского пола — 52,8 см. В итоге конечный рост у подростков, лечившихся в среднем 7 лет (от 6,0 до 10,5 мальчики и от 5,0 до 10,0 лет девочки), составил 174,7 см (173,0–179,5 см) у юношей и 162,3 см (158,8–164,4 см) у девушек. При этом разница между показателями прогнозируемого конечного роста и достигнутым ростом составила всего 1,3 см у юношей и 0,8 см у девушек. А у некоторых подростков SDS конечного роста даже превосходила SDS генетически прогнозируемого роста.

Метаболический эффект соматропина приводил к повышению общего тонуса организма, увеличению мышечной силы, нормализации липидного и углеводного обменов, повышению сердечного выброса и улучшению минерализации костей.

В течение последних 30 лет в Институте детской эндокринологии ГНЦ РФ ФГБУ «НМИЦ эндокринологии» Минздрава России ведется системная работа по изучению функциональной системы, регулирующей синтез и секрецию СТГ, включая регуляторные белки гипоталамуса, гормоны гипофиза, ростовые и транскрипционные факторы, а также широкий спектр метаболических и системных эффектов СТГ в различные возрастные периоды.

Начало 1990-х гг. ознаменовалось началом активного изучения причин гипофизарного нанизма. Одной из первых в мире, совместно с Yamashita S. и другими японскими учеными., Фофанова О.В., изучая множественный дефицит гормонов аденогипофиза (МДГА), выявила дефекты в генах PIT1 и PROP1 [7–16]. Эти исследования были опубликованы в ведущих зарубежных журналах, признаны мировым научным сообществом и многократно цитируются по сей день. При исследовании гена гормона роста (GH1) и гена рилизинга гормона роста (GHRH) как причины изолированного дефицита гормона роста (ИДГР) у детей были выявлены различные молекулярные дефекты (делеции, сдвиги рамки считывания, нонсенс и миссенс-мутации, а также сплайсинг-мутации) [17–19].

Большой вклад в изучение причин низкорослости внесли исследования Тюльпакова А.Н. [6] и Рубцова П.М. При их непосредственном участии активно изучался рецептор гормона роста (GHR), ответственный за развитие синдрома Ларона [20][21]. Одновременно с этим Пятушкиной Г.А. проводились исследования полиморфизма гена рецептора гормона роста (GHR) [22].

Молекулярно-генетические исследования различных причин низкорослости, проведенные в конце 90-х гг. XX века, привели к формулированию понятия клинической гетерогенности и разного ростового эффекта на применение гормона роста.

Изучение синдромальной патологии роста продолжалось, и в 1997 году в мире был открыт SHOX-ген, локализованный на коротком плече Х или Y-хромосомы, ответственный за развитие низкорослости при синдромах Шерешевского-Тернера и Лери-Вейлла [23]. Исследования Шандина А.Н. выявили мутации этого гена у 1,2% детей с идиопатической низкорослостью [24][25]. Эти данные легли в основу детального изучения эффективности терапии рГР при синдроме Шерешевского-Тернера Панкратовой М.С. [26].

Проведенные исследования послужили основой для последующего изучения проблемы низкорослости у детей А.В. Витебской. Большой интерес представляло изучение причин идиопатической «семейной» низкорослости и синдромальных форм задержки роста, а также выработка показаний для назначения терапии рГР при подобных состояниях [27]. Первые исследования, рассматривающие возможность развития идиопатического СТГ-дефицита вследствие аутоиммунного гипофизита, проводились еще О.В. Фофановой и датируются 1993 годом [28].

Изучение причин низкорослости у детей развивалось быстрыми темпами, привлекая все большее внимание медицинского сообщества, и в 2005 г. О.А. Чикулаева завершила наши исследования, касающиеся молекулярно-генетических особенностей врожденной соматотропной недостаточности у детей в рамках европейской программы GeNeSIS. Были получены новые данные о встречаемости различных вариантов молекулярно-генетических дефектов при соматотропной недостаточности у детей в российской популяции [29].

Не только молекулярные основы, но и клинические проявления СТГ-дефицита были предметом изучения детских эндокринологов. В ходе изучения метаболических эффектов гормона роста Н.Н. Волеводз было выявлено, что у 58% детей с СТГ-дефицитом выявляется гиперхолестеринемия, остеопороз — у 36%, остеопения — у 47% детей, также было отмечено снижение размеров сердца относительно возраста [30].

За комплексные достижения в изучении нарушений секреции гормона роста при различных заболеваниях эндокринной системы у детей академику РАН, профессору В.А. Петерковой в 2004 г. была вручена национальная премия лучшим врачам России «Призвание» в номинации «За создание нового направления в медицине».

В 2005 г. на основании анализа зарубежных исследований и отечественного опыта применения гормона роста был разработан и опубликован первый Национальный консенсус по лечению соматотропной недостаточности [31], который позже был актуализирован и является по настоящее время основным документом, регламентирующим диагностику и лечение детей и подростков с соматотропной недостаточностью [32].

На протяжении последних 15 лет все дети с доказанной соматотропной недостаточностью обеспечиваются соматотропином за счет средств федерального бюджета. Проведенный М.В. Воронцовой медико-экономический анализ различных аспектов лечения детей с гипофизарным нанизмом доказал, что терапия детей в рамках государственной программы высокозатратных нозологий является клинически и экономически эффективной [33][34].

В последние годы стремительно развиваются знания об этиологических, патогенетических и молекулярно-генетических основах низкорослости. Понимание особенностей органогенеза гипофиза, физиологической роли СТГ в регуляции роста привело к открытию ряда моногенных форм гипопитуитаризма, характеризующихся уникальной клинической картиной. В настоящее время известен целый ряд генов, ответственных за закладку и развитие структурных элементов гипофиза и синтез гормонов, таких как: PROP1, POU1F1, PTX, LIF, HESX1, RPX3, LHX3, LHX4, SOX2, SOX3, GLI2, OTX2, GH1, GHPHR, RNPC3, IGSF1, TBL1X, TBX19, PCSK1, TCF7L1, PROKR2, FGFR1, FGF8, KAL1, ROBO1, ARNT2, PNPLA6, KCNQ1. С каждым годом эта информация обновляется и дополняется.

Накопленные данные о молекулярно-генетических основах синдрома низкорослости у детей помогают раскрывать причины редких заболеваний и назначать для их лечения персонифицированную терапию.

В настоящее время в зависимости от ситуации существует большой спектр генетических исследований для поиска причины низкорослости. Есть возможность изучить отдельный ген, панель генов (например, панель «Гипопитуитаризм», включающую 23 наиболее часто встречающихся гена, ответственных за развитие гипофизарного нанизма), а также провести полноэкзомное секвенирование. Благодаря поддержке Национальной благотворительной программы помощи детям с эндокринными заболеваниями «Альфа-Эндо» молекулярно-генетическое исследование при необходимости доступно всем детям из любого региона страны в лаборатории генетики моногенных эндокринных заболеваний ГНЦ РФ ФГБУ «НМИЦ эндокринологии» Минздрава России.

В дифференциальной диагностике различных форм низкорослости используется кариотипирование и хромосомный микроматричный анализ, особенно в случаях синдромальной низкорослости.

Результаты молекулярно-генетического исследования 625 детей выявили у 20,5% генетическую причину гипопитуитаризма. Чаще всего встречались варианты в гене PROP1 (44% — 56 пациентов, из них 16 сибсов), GH1 (13% — 17 пациентов), POU1F1 (7% — 9 пациентов) GLI2 (7% — 9 пациентов), GHRHR (5,5% — 7 пациентов), IGSF1 (4% — 5 пациентов), HESX1 (3% — 4 пациента) и др. (рис. 2).

**Figure:**
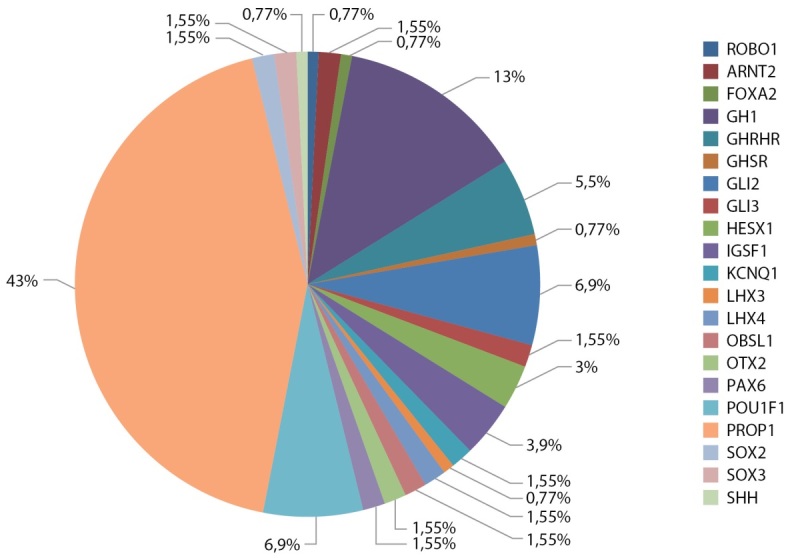
Рисунок 2. Генетическая разнородность гипопитуитаризма.

Данное многолетнее исследование, в котором принимали участие детские эндокринологи из различных регионов Российской Федерации, позволило найти причину многих редких моногенных форм гипопитуитаризма. Так, только за последние 5 лет были выявлены 52 пациента с редкими синдромальными формами низкорослости (2 ребенка с артрогриппозом с холестазом (VPS33B), 1 — с синдромом Саньяд-Сакати (TBCE), 2 — с синдромом Рубинштейна-Тейби (CREBBP), 1 — с синдромом Робинов (DVL1), 3 — с синдромом Ройфмана (RNU4ATAC), 5 — с «3-М» синдромом (CUL7), 3 — с синдромом Аарского-Скотта (FGD1), 2 — с синдромом Плавающей гавани (SRCAP), 5 — с синдромом Секкеля (CENPJ, CEP63, RBBP8), 2 — с анемией Фанкони (ATR), 8 — с дефектом гена аггрекана (ACAN) и др.)

Таким образом, медико-генетические исследования последних лет существенно расширяют наши знания о причинах синдромальной низкорослости.

Планирующиеся исследования направлены на изучение редких моногенных форм гипопитуитаризма у детей и выявление гено-фенотипических корреляций при этой патологии для разработки персонализированного подхода к лечению и наблюдению.

## СТГ-ДЕФИЦИТ У ВЗРОСЛЫХ

Гормон роста синтезируется в течение всей жизни и обладает не только рост-стимулирующим эффектом. Он определяет величину и силу мышечной массы, массу и минеральную плотность кости [35], регулирует жировой и углеводный обмен, объем внеклеточной жидкости [36], влияет на различные функции ЦНС, обладает влиянием на иммунитет. Как анаболический гормон он необходим на протяжении всей жизни взрослого человека. Продолжительность жизни больных с гипофизарной карликовостью без лечения на 20–30 лет короче общепопуляционной.

Первые плацебо-контролируемые долгосрочные исследования, посвященные терапии СТГ-дефицита у взрослых пациентов, датируются 1989 г. [37][38]. В Российской Федерации изучение влияния дефицита ГР у взрослых пациентов с соматотропной недостаточностью началось в конце 90-х гг. XX века.

Исследования Е.В. Нагаевой включали изучение композиционного состава тела, подбор оптимальной дозы препарата рГР у взрослых пациентов, особенности течения гипопитуитаризма [39][40].

К 2005 г. были подведены итоги и доказана эффективность и безопасность применения рГР у лиц старше 18 лет (О.Б. Безлепкина и соавт.) [41–43].

Клиническая картина дефицита СТГ у взрослых проявляется, помимо карликовости, висцеральным ожирением, гиперлипидемией, дислипидемией, характерно развитие нарушений углеводного обмена (инсулинорезистентность, нарушение толерантности к глюкозе). Заместительная терапия рГР с использованием метаболической дозы (в 7–10 раз меньше ростостимулирующей дозы) помогает нормализовать показатели липидного обмена и улучшить минеральную плотность костной ткани. Установлено, что ГР обладает выраженным эффектом на процессы ремоделирования костной ткани. Денситометрические исследования выявили прогрессирующую остеопению и снижение процессов остеосинтеза при соматотропной недостаточности [35]. Доказано, что у лиц, получающих заместительную терапию рГР, нормализуются процессы ремоделирования костной ткани [44].

Существуют многочисленные данные о повышенном риске сердечно-сосудистых заболеваний и смертности у взрослых с СТГ-дефицитом (диастолическая дисфункция миокарада, нарушение проводимости, сократимости, уменьшение массы миокарда, снижение фракции выброса) [45][46]. А на фоне же лечения рГР происходит улучшение функции миокарда. Эти данные послужили основой для использования р-ГР при миокардитах, миокардиодистрофиях и сердечной недостаточности с положительным эффектом [47].

## ОБЕСПЕЧЕНИЕ ПАЦИЕНТОВ ГОРМОНОМ РОСТА В РФ

На протяжении последних 15 лет в Российской Федерации все дети с доказанной соматотропной недостаточностью (гипофизарным нанизмом) бесплатно обеспечиваются соматотропином, который входит в перечень централизованно закупаемых за счет средств федерального бюджета лекарственных средств (действующий документ — Распоряжение Правительства РФ №2738-р от 10.12.2018 г.).

Государственная поддержка пациентов с дефицитом гормона роста масштабно началась в конце 2008 г., когда была создана программа «Высокозатратные нозологии» (ВЗН), первоначально она включала 7 нозологий, в том числе «гипофизарный нанизм». Спустя некоторое время — в 2019 г. — программа включала в себя уже 12 высокозатратных нозологий, а в 2020-м — 14 нозологий. При этом гипофизарный нанизм всегда был и остается неотъемлемой частью программы, что позволяет полноценно обеспечивать всех детей с соматотропной недостаточностью необходимым лечением и анализировать эффективность терапии рГР.

Сотрудниками Минздрава России совместно с экспертами — детскими эндокринологами Института детской эндокринологии — в целях осуществления государственных закупок проводится ежегодная экспертиза всех случаев соматотропной недостаточности у детей в России с последующим обеспечением их гормоном роста. С 2023 г. финансирование этих закупок стало проводиться при участии фонда поддержки детей с тяжелыми жизнеугрожающими и хроническими заболеваниями, в том числе редкими (орфанными) заболеваниями «Круг добра».

Начиная с 2022 г. взрослые с впервые выявленным СТГ-дефицитом, а также молодые люди старше 18 лет с соматотропной недостаточностью после проведения ретестирования и подтверждения соматотропной недостаточности имеют возможность обеспечения рГР в рамках программы «14 высокозатратных нозологий». Не подлежат ретестированию подростки с подтвержденной молекулярно-генетической причиной заболевания и рядом других состояний, указанных в Консенсусе по диагностике соматотропной недостаточности [32].

Реестр пациентов с соматотропной недостаточностью на конец 2023 г. включал в себя 4639 пациентов (4520 детей и 119 взрослых).

## ГОРМОН РОСТА ПРИ ЗАБОЛЕВАНИЯХ, НЕ СВЯЗАННЫХ С СОМАТОТРОПНОЙ НЕДОСТАТОЧНОСТЬЮ

Новые достижения привели к расширению клинического применения гормона роста, что открыло новые возможности в лечении других форм низкорослости. Это важный прогресс, который нельзя не отметить.

Еще в 1983 г. на Международной конференции по применению гормона роста возник вопрос о необходимости его использования у низкорослых детей, не имеющих СТГ-дефицита. Доступность рГР позволила проводить исследования его эффективности при различных негипофизарных причинах низкорослости. Целью терапии низкорослых детей без соматотропной недостаточности является увеличение скорости роста и нормализация линейного роста без воздействия на основные причины их заболевания. Рекомбинатный ГР применяется при различных состояниях, таких как синдромальная низкорослость (например, синдромы Шерешевского-Тернера, Сильвера-Рассела, Прадера-Вилли, Де Груши), хроническая почечная недостаточность, длительная терапия глюкокортикоидами в анамнезе, внутриутробная задержка роста, идиопатическая низкорослость, ювенильный идиопатический артрит, заболевания кроветворной системы, нейрофиброматоз, муковисцидоз и др. При всех этих заболеваниях низкорослость вызвана не соматотропной недостаточностью, для таких пациентов характерен дозозависимый характер эффективности ГР и проводится очень внимательный мониторинг.

В Российской Федерации, кроме соматотропной недостаточности, у детей и взрослых лечение гормоном роста одобрено при ХПН, синдроме Шерешевского-Тернера, синдроме Прадера-Вилли, низкорослости вследствие задержки внутриутробного развития.

## СИНДРОМ ШЕРЕШЕВСКОГО-ТЕРНЕРА (СШТ)

Терапия рГР применяется для увеличения роста у пациенток с СШТ, что позволяет им достичь роста, близкого к нормальному. В среднем использование рГР увеличивает рост пациенток на 5–8 см за 5–7 лет терапии, что, безусловно, демонстрирует его эффективность. Возраст начала и длительность лечения рГР влияет на конечный рост, поэтому очень важно начинать терапию как можно раньше, чтобы предотвратить замедление скорости роста, характерное для девочек с СШТ первых лет жизни. Оптимальный возраст начала терапии рГР при СШТ не определен, в большинстве стран лечение инициируют после 4 лет. Начальная доза рГР при СШТ составляет 0,05 мг/кг в сутки с последующей коррекцией в зависимости от ростового эффекта. При своевременно начатом лечении рГР большинство исследователей рекомендуют начинать лечение эстрогенами с 9–12 лет [26, 48].

В 2023 г. фонд поддержки детей с тяжелыми жизнеугрожающими и хроническими заболеваниями, в том числе редкими (орфанными) заболеваниями, «Круг добра» взял на себя обеспечение соматропином пациенток с генетически подтвержденным синдромом Шерешевского-Тернера. Установлены следующие критерии для начала лечения: возраст от 4 до 14 лет; задержка роста до начала лечения (SDS роста для популяции ≤2,0) и рентгенологически установленный костный возраст меньше 13 лет. В настоящее время происходит экспертиза заявок и включение детей с СШТ в программу лечения.

## ЗАДЕРЖКА ВНУТРИУТРОБНОГО РАЗВИТИЯ (ЗВУР)

В отдельную нозологию выделена низкорослость, обусловленная задержкой внутриутробного развития. Первое крупное рандомизированное исследование продолжительного применения рГР у этой когорты детей датируется 1990 г. Наличие ЗВУР в анамнезе связывают с повышенным риском развития низкорослости, раним началом с быстрым прогрессированием полового созревания, нейро-когнитивными дисфункциями, метаболическими изменениями (сниженная плотность костей, нарушения углеводного обмена, дислипидемии), повышенным риском сердечно-сосудистых заболеваний в более позднем возрасте. У подавляющего большинства детей со ЗВУР наблюдается спонтанное догоняющее развитие в первые годы жизни.

В Институте детской эндокринологии ГНЦ РФ ФГБУ «НМИЦ эндокринологии» Минздрава России в течение последних 20 лет проводятся наблюдение и при необходимости лечение низкорослых детей со ЗВУР. Е.В. Нагаевой изучен большой спектр клинических, гормональных, метаболических особенностей у пациентов со ЗВУР [49]. Многолетний опыт применения рГР у детей со ЗВУР доказал, что предикторами эффективности терапии являются доза препарата и сроки инициации терапии (как и при СТГ-дефиците предпочтительным является раннее начало терапии) [50].

Кроме того, гормон роста применяется в терапии низкорослости, обусловленной длительной терапией глюкокортикоидами у детей, для которых характерна супрессия активности центрального и периферического звена рострегулирующей системы. Совместно с клиникой Сеченовского Университета изучался вопрос применения рГР у детей с ревматическими заболеваниями, была показана эффективность и безопасность лечения этой когорты детей [51].

Хроническая почечная недостаточность (ХПН), характеризующаяся развитием уремии и связанной с ней потерей баланса между ИФР1 и ИФРСБ-3, также приводит к задержке роста у детей [52][53], при этом наиболее выраженную низкорослость имеют дети с врожденными нарушениями [54]. Зарубежный и наш опыт свидетельствует об эффективности и безопасности лечения этой группы детей, низкорослость при ХПН у детей — одно из первых, не обусловленных СТГ-дефицитом состояний, одобренных для лечения рГР.

Перспективным направлением считалось и считается применение рГР в геронтологии. Первые сообщения об улучшении композиционного состава тела у пожилых мужчин с низкими уровнями ИФР-1 в плазме в ответ на инъекции рГР, опубликованное в 1990 г. [55], привлекло огромное внимание к рГР как к «антивозрастному» средству. Многие исследователи полагают, что изучение роли СТГ в процессах старения, механизме взаимодействия с факторами окружающей среды приведут к новым знаниям, важным как для индивидуального, так и для общественного здоровья [56].

Гормон роста также играет роль в поддержании скелетной массы у взрослых. Он стимулирует хондроциты и остеобласты, являющиеся клетками-предшественниками, участвующими в пролиферации и дифференцировке. В связи с описанными эффектами СТГ на костную ткань, предпринимались попытки назначения рГР людям с остеопорозом без СТГ-дефицита. Gonnelli S. и др. в своем плацебо-контролируемом исследовании показали, что у женщин в постменопаузальный период лечение рГР при остеопорозе увеличивает обмен костной ткани. Комбинированное лечение рГР и кальцитонином в течение 2 лет повышает и поддерживает МПК в позвоночнике и лучевой кости, снижая МПК бедренной кости [57]. В другом исследовании Barake M. с соавт. показали, что терапия рГР не всегда улучшает плотность костной ткани у женщин с возрастной потерей костной массы, но в большинстве случаев снижает риск переломов [58].

## ПЕРСПЕКТИВЫ: ПРЕПАРАТЫ СТГ ДЛИТЕЛЬНОГО ДЕЙСТВИЯ

Необходимость ежедневного подкожного введения рГР в течение многих лет часто является препятствием к оптимальному соблюдению режима лечения. Многие годы исследователи были сосредоточены на разработке препаратов СТГ длительного действия с целью продления периода полувыведения молекулы СТГ и снижения частоты введения.

В 2013 г. Европейским агентством лекарственных средств (EMA) был одобрен зарегистрированный ранее в Южной Корее депо — препарат LB03002 для еженедельного подкожного введения (www.ema.europa.eu/en/medicines/human/EPAR/somatropin-biopartners). Однако разрешение на его использование было отозвано ввиду того, что он не поступил в продажу в течение 3 лет.

В настоящее время активно проводятся исследования минимум восьми препаратов, в пяти случаях идет исследование III фазы [59]. При этом, в отличие от рГР, представляющего собой один пептид массой 22 кДА, производимый аналогичным всеми фармакологическими компаниями, каждый из препаратов длительного действия представляет собой отдельный химический субстрат, со своим профилем фармакокинетики и фармакодинамики. Mameli С. и соавт. в 2023 г. представили крупный метаанализ всех изучаемых препаратов СТГ длительного действия с 2012 по 2022 гг. и пришли к выводу о том, что в отношении эффективности и безопасности все доступные препараты были аналогичны рГР для ежедневного использования [60].

Таким образом, мы являемся свидетелями начала и широкого внедрения в клиническую практику лечения дефицита гормона роста как у детей, так и у взрослых. Казавшееся когда-то невероятным, становится рутинной практикой каждого детского эндокринолога. Развитие молекулярной генетики расширяет наше понимание основ гипопитуитаризма. Кто знает, возможно, следующие 10–20 лет ознаменуются появлением и генно-инженерной терапии, что позволит говорить о полной ликвидации карликовости.

## ДОПОЛНИТЕЛЬНАЯ ИНФОРМАЦИЯ

Источники финансирования. Работа проведена в рамках темы госзадания 123021000045–4 «Генетическая персонификация редких вариантов задержки роста и полового развития у детей».

Конфликт интересов. Авторы декларируют отсутствие явных и потенциальных конфликтов интересов, связанных с содержанием настоящей статьи.

Участие авторов. Райкина Е.Н., Панкратова М.С., Нагаева Е.В. — аналитическая работа и подготовка финальной версии статьи; Безлепкина О.Б., Петеркова В.А., Дедов И.И. — редактирование текста, внесение ценных замечаний. Все авторы одобрили финальную версию статьи перед публикацией, выразили согласие нести ответственность за все аспекты работы, подразумевающую надлежащее изучение и решение вопросов, связанных с точностью или добросовестностью любой части работы.
